# Georgia Medical Association

**Published:** 1873-02

**Authors:** 


Georgia Medical Association.—Being earnestly desirous that the approaching meeting of the Georgia Medical Association shall represent fully the medical profession of the State, we may be allowed to urge not only every member of the Association to attend, but to bring with him the medical friends in his vicinity. It is believed that this Association is about to enter upon a new and higher future, and will be well worthy of the support and fostering aid of the profession.

There is no more central or accessible point than this city, and we are sure that our medical friends will not go away dissatisfied with their reception. Come one, come all, and let us put the Georgia Medical Association upon as high a basis as any other medical organization in the land.

We regret that irregularities have occurred in connection with the issue of the Journal for several months. We are assured, however, by the publishers and proprietors, "Herald Publishing Company," that this will not occur in the future.

We have additional arrangements made for other European correspondence, which will add *much* to this already very interesting feature of the Journal.

